# Supplementary material for: The Impact of Next-Generation Sequencing Added to Multiplex PCR on Antibiotic Stewardship in Critically Ill Patients with Suspected Pneumonia
Source: Diagnostics (Basel). 2024 Jun 29;14(13):1388. doi: 10.3390/diagnostics14131388 (PMC11241116; doi:10.3390/diagnostics14131388)
Supplement: Supplementary file 1 [file diagnostics-14-01388-s001.zip › diagnostics-3074795-supplementary.pdf]

BAL culture	BioFire Pneumonia Panel	RPIP
Acinetobacter baumannii (n =1)	*Acinetobacter baumannii* (n = 2)	*Acinetobacter baumannii* (n = 1)
Acinetobacter radioresistens (n =1)	*Escherichia coli* (n = 2)	*Achromobacter xylosoxidans* (n = 2)
Aspergillus species (n = 3)	*Haemophilus influenzae* (n = 2)	Adenovirus C (n = 1)
Escherichia coli (n = 1)	*Klebsiella oxytoca* (n =1)	Coronavirus HUK1 (n = 1)
Haemophilus influenzae (n =1)	*Klebsiella pneumoniae* (n = 3)	*Escherichia coli* (n = 2)
Klebsiella oxytoca (n =1)	*Moraxella catarrhalis* (n = 1)	*Haemophilus influenzae* (n = 1)
Klebsiella pneumoniae (n =2)	Proteus species (n = 1)	*Klebsiella oxytoca* (n =1)
Legionella pneumophila (n = 1)	*Pseudomonas aeruginosa* (n= 6)	*Klebsiella pneumoniae* (n = 3)
Mycobacterium abscessus (n = 1)	Respiratory syncytial virus (n = 1)	*Mycobacterium avium* complex (n = 1)
Mycobacterium avium complex (n = 5)	Rhinovirus (n = 1)	*Mycobacterium kansasii* (n = 1)
Proteus mirabilis (n = 1)	*Staphylococcus aureus* (5)	*Proteus mirabilis* (n = 1)
*Pseudomonas aeruginosa (*n= 4)		*Pneumocystis jirovecii* (n = 1)
Rhizopus species (n = 1)		*Pseudomonas aeruginosa* (n = 6)
Staphylococcus aureus (3)		SARS-CoV-2 (n = 7)
Stenotrophomonas maltophilia (n =1)		*Staphylococcus aureus* (n = 2)
		Stenotrophomonas maltophilia (n =3)
		Haemophilus parainfluenzae (n = 1)
		Streptococcus anginosus group (n = 4)
		Actinomyces species (n = 4)
		Bacteroides fragilis (n = 1)
		Capnocytophaga species (n = 7)
		Corynebacterium species (n = 5)
		Cytomegalovirus (n = 4)
		Delftia species (n = 1)
		Dialister species (n = 2)
		Dolosigranulum species (n = 1)
		Ebstein-Barr Virus (n = 5)
		Eikenella species (n = 1)

	Fusobacterium species (n = 2)
	Herpes-simplex virus-1 (n = 2)
	Human herpes-virus 6 (n = 2)
	Parvimonas species (n = 2)
	Prevotella species (n = 10)
	Pseudomonas fluorescens (n = 5)
	Rothia species (n = 8)
	Slackia species (n = 1)
	Trophyerema whipplei (n = 1)
	Ureaplasma urealyticum (n = 1)
	Veillonella species (n = 8)

Supplement Table S1. Organisms identified by each diagnostic method. Each is listed in alphabetical order and columns should not be compared. Organisms in blue text are those with unclear pathogenicity.

Routine Diagnostics Result	RPIP Result	New/Additional Organisms Identified	Potential change in Antibiotic Management
Culture and/or mPCR positive	No new Organisms (10)	--	No change
	Positive (24)	EBV	No change
		Eikenella corrodens, Pseudomonas fluorescens	No change (patient culture positive for Pseudomonas aeruginosa, antibiotics for this would cover additional RPIP organisms)
		Pseudomonas fluorescens	No change (patient culture positive for Pseudomonas aeruginosa, antibiotics for this would cover additional RPIP organism)
		Veillonella parvula, Ureaplasma urealyticum, Streptococcus constellatus	No change (patient culture positive for S. aureus and E. faecalis, antibiotics for these would cover additional RPIP organisms)
		Rothia mucilaginosa, Prevotella melaninogenica, Capnocytophaga sputigena	No change (patient culture positive for Acinetobacter baumannii, antibiotics for these would cover additional RPIP organisms)
		Prevotella melanin, Streptococcus mitis, Actinomyces graevenitzii,	Add anaerobic coverage or Beta-lactam (patient culture positive for MAC and Viridans streptococcus)

		Veillonella parvula,<br>Campylobacter consicus	
		Mycobacterium avium complex,<br>Rothia mucilaginosa,<br>Actinomyces graenvenitzii,<br>Prevotella melaningogenica,<br>Veillonella parvula,<br>Streptococcus mitis,<br>Campylobacter coniscus	Add treatment for MAC<br>Add anaerobic coverage or Beta-lactam (patient only culture positive for Viridans streptococcus)
		Achromobacter xylosox,<br>Bacteroides fragilis,<br>Corynebacterium striatum,<br>Parvimonas micra, Prevotella melani, Streptococcus constellatus, Fusobacterium nucleatum, Pseudomonas fluorescens	No change (Patient culture positive for P. aeruginosa, and anti-Pseudomonal therapy would cover the additional RPIP organisms
		Pseudomonas fluorescens	No change (patient culture positive for Pseudomonas aeruginosa)
		EBV, HHV6	Add antiviral for HHV6
		EBV, HHV6, CMV<br>Corynebacterium striatum	Add antiviral for CMV and HHV6
		HSV-1, K. oxytoca, Veillonella parvula	Add antiviral for HSV

		Rothia mucilaginosa, Streptococcus mitis, Veillonella parvula, Dialister pneumosintes, Prevotella melanino, Haemophilus parainfluenzae, Campylobacter coniscus	No change<br>(patient was culture positive for K. pneumoniae and H. influenzae, antibiotics for these organisms would cover the additional ones identified by RPIP)
		Prevotella melaningogenica, Rothia mucilaginosa, Campylobacter coniscus	No change<br>(patient culture positive for Staphylococcus aureus and Lactobacillus)
		Corynebacterium striatum, Stenotrophomonas spp., Pseudomonas fluorescens	Add antibiotic for Stenotrophomonas spp.<br>(patient already culture positive for P. aeruginosa, no additional therapy for P. fluorescens).
		Stenotrophomonas spp., Achromobacter xylosoxidans, Klebsiella pneumoniae, Corynebacterium striatum, Delftia spp., Pseudomonas fluorescens	Add antibiotic for Stenotrophomonas spp.<br>(patient culture positive for Acinetobacter baumannii, antibiotics for this organism would cover additional organisms identified by RPIP)
		EBV, CMV, Enterococcus faecium, Corynebacterium spp.	Add antiviral for CMV<br>Add antibiotic for E. faecium
		EBV, CMV, Enterococcus faecium	Add antiviral for CMV
		HHV-6	Add antiviral

		Dolosigranulum pignum	Add Gram-positive coverage (patient only positive for Covid)
		Human adenovirus	No change
		Rothia mucilaginosa, Acinetobacter pitii, Stenotrophomonas maltophila	Add antibiotic for Stenotrophomonas spp. (patient culture positive for Acinetobacter baumannii, antibiotics for this organism would cover additional organisms identified by RPIP)
		Staphylococcus aureus, Prevotella melanino, Rothia mucilaginosa	No change (patient culture positive for methicillin resistant S. aureus)
		Rothia mucilaginosa, Prevotella pleuritidis, Dialister pneumosintes, Parvimonas micra, Veillonella parvula, Actinomyces israelii, Slackia exigua, Campylobacter consicus, Fusobacterium nucleatum	Add high dose penicillin for A. israelii (patient culture positive for H. influenzae and Streptococcus spp., antibiotics for this would cover additional RPIP organisms)
Culture and mPCR negative	Negative (0)	--	No change
	Positive (4)	Pneumocystis jirovecii	Add trimethoprim-sulfamethoxazole and possible prednisone
		Rothia mucilaginosa	Add B-lactam
		Pseudomonas aeruginosa	Add anti-Pseudomonal
		Escherichia coli	Add Gram-negative coverage

Supplement Table S2. The potential impact of RPIP results on antibiotic therapy for the 38 cases of suspected pneumonia.
